# Effects of Ramadan and Non-ramadan Intermittent Fasting on Gut Microbiome

**DOI:** 10.3389/fnut.2022.860575

**Published:** 2022-03-22

**Authors:** Seyedeh Neda Mousavi, Elham Rayyani, Javad Heshmati, Ronia Tavasolian, Mehran Rahimlou

**Affiliations:** ^1^Department of Nutrition, School of Medicine, Zanjan University of Medical Sciences, Zanjan, Iran; ^2^Zanjan Metabolic Diseases Research Center, Zanjan University of Medical Sciences, Zanjan, Iran; ^3^Department of Clinical Nutrition, School of Nutritional Sciences and Dietetics, Tehran University of Medical Sciences (TUMS), Tehran, Iran; ^4^Songhor Healthcare Center, Kermanshah University of Medical Sciences, Kermanshah, Iran; ^5^Faculty of Clinical Science and Nutrition, University of Chester, Chester, United Kingdom; ^6^Department of Nutrition, School of Medicine, Zanjan University of Medical Sciences, Zanjan, Iran

**Keywords:** fasting, intermediate fasting, Ramadan, gut microbiome, review

## Abstract

**Background:**

In recent years, intermittent fasting (IF) has gained popularity in the health and wellness in the world. There are numerous types of IF, all of which involve fasting periods that last longer than an overnight fast and involve limited meal time-windows, with or without calorie restriction. The objective of this review is to summarize the current evidence for the effects of Ramadan and non-Ramadan IF on gut microbiome.

**Methods:**

We explored PubMed, Scopus, Web of Science, and Google Scholar according to the PRISMA criteria (Preferred Reporting Items for Systematic Reviews and Meta-Analysis). Animal and human studies were screened and reviewed separately by two researchers.

**Results:**

Twenty-eight studies were selected after screening. Some of the studies were performed on animal models and some on humans. The results of these studies indicate a significant shift in the gut microbiota, especially an increase in the abundance of *Lactobacillus* and *Bifidobacteria* following fasting diets. The results of some studies also showed an increase in the bacterial diversity, decrease inflammation and increased production of some metabolites such as short-chain fatty acids (SCFAs) in individuals or samples under fasting diets. Moreover, Ramadan fasting, as a kind of IF, improves health parameters through positive effects on some bacterial strains such as *Akkermansia muciniphila* and *Bacteroide*. However, some studies have reported adverse effects of fasting diets on the structure of the microbiome.

**Conclusion:**

In general, most studies have seen favorable results following adherence from the fasting diets on the intestinal microbiome. However, because more studies have been done on animal models, more human studies are needed to prove the results.

## Introduction

People can calorically restrict while feeling hungry, and this approach has already been demonstrated in various mammalian species to enhance life span, increase numerous physiological indicators, and lower metabolic parameters for chronic illness (1, 2). There are numerous types of intermittent fasting (IF), all of which involve fasting periods that last longer than an overnight fast and involve limited meal time-windows, with or without calorie restriction (3, 4).

The Islamic lunar calendar’s ninth month, Ramadan, is 11–12 days shorter than the Gregorian solar calendar. This indicates that the month of Ramadan revolves around the four seasons and the 12 months of the year. Fasting during Ramadan is an obligatory duty for all healthy adult Muslims, as stated in the Holy Quran where ALLAH says, “O you who believe, fasting is prescribed for you as it was prescribed for those before you, that you may develop God-consciousness” (Surat Al-Baqarah 2:183). Ramadan fasting is one of the most common types of fasting diets in which millions of Muslims around the world do not receive any food or drink for a daily time varies between 12 and 22 h (mean 12–14 h), depending on the geographical location and season during a special month for a month. Ramadan also spelled Ramazan, Ramzan, Ramadhan, or Ramathan, is the ninth month of the Islamic calendar, observed by Muslims worldwide as a month of fasting (sawm), prayer, reflection and community (5). According to Islamic law, during the days of Ramadan, healthy adults must fast at certain times of the day, while fasting is not required for premature children, the elderly, the sick, and pregnant and lactating women (6) #42; (7) #32.

In addition to Ramadan fasting diets, in recent years, there has been an increased interest in following modified fasting diets aimed at weight loss or the management of some chronic diseases among people in different countries (8). IF have greatly increased in recent decades as weight loss and some other metabolic benefits (9). The effectiveness of these diets in weight loss or management of metabolic parameters has varied depending on the type and duration of fasting diets (10).

The human gastrointestinal microbiome, which contains millions of organisms can be affected by various environmental factors such as diet. On the other hand, various studies have shown that adverse changes in the intestinal microbiome can be associated with the development of various chronic diseases (11). Some findings have revealed that fasting diets can also cause changes in the microbiome (12, 13).

The objective of this review is to summarize the current evidence for the effects of Ramadan and non-Ramadan IF on gut microbiome. We first review the evidence from pre-clinical studies to provide a background on the purported mechanisms by which fasting diets induces changes in gut microbiome and then focused on human studies.

## Methods

**FIGURE 1:**
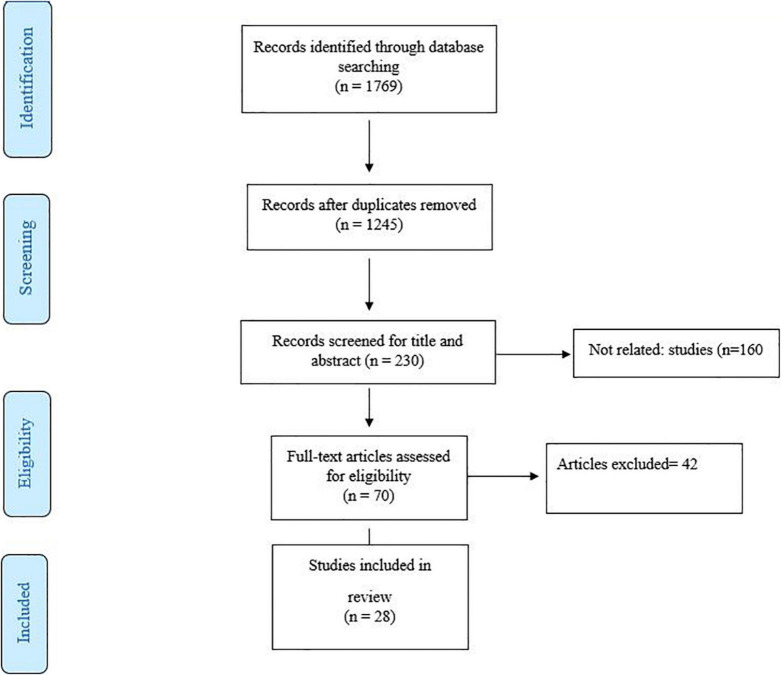
Flow diagram of study.

The PubMed, Web of Science, Scopus, and Google Scholar databases were searched from their inception until December 2021 according to the PRISMA criteria (Preferred Reporting Items for Systematic Reviews and Meta-Analysis). We used from the keywords included “gut microbiome” OR “Fecal microbiota” OR “Gut microbial profile” OR “Gut microbiota” OR “gut flora” OR “intestinal flora” OR “intestinal microbiota” in combination with Fasting OR “Intermittent fasting” OR “Ramadan Fasting” OR “Islamic fasting.” Additional items were added after examining the referenced articles (Figure 1). Two authors (MR and SM) independently assessed the abstract and full text of the articles, and animal and human studies, which evaluated the effect of different types of fasting on the microbiome, were screened (Table 1). Disagreements were resolved by consensus. Studies were included in this review article if the following conditions were met: (1) animal and human studies investigating the effect of fasting diets on the gut microbiota, (2) in order to evaluating the gut microbiota alterations in various fasting conditions and probable mechanisms in improving overall metabolic health, types of fasting regimens were classified into two main subgroups (time restricted fasting including Ramadan fasting and 8/16 h fasting program and calorie restricted fasting including alternate day fasting, water only fasting and the Buchinger program). Studies were excluded if the main text was not available or was not in the English language. Reviews, protocols, conference papers and case reports were also excluded. Therefore, only original researches with original data on animal models or human patients exploring any kind of fasting regimes on gut microbiota were included in the present study.

**TABLE 1:** Table of inclusion and exclusion criteria following the PICOS approach^1^.

PICOS	Inclusion and exclusion criteria	Data extraction
Participants	Adult population’s ≥18 and ≤65 years or animal samples were included.	Age, sex, gender, sample size, location, inclusion and exclusion criteria
Intervention	Types of fasting regimens were classified into two main subgroups (time restricted fasting including Ramadan fasting and 8/16 h fasting program and calorie restricted fasting including alternate day fasting, water only fasting and the Buchinger program).	Types of fasting regimens, fasting duration
Comparators	Only studies with control group were included, participants or animal models with normal diet.	Type of comparator, compliance
Outcomes	Changes in the gut microbiota.	Outcomes measured, evaluation methods and side effects.
Study design	Studies were excluded if the main text was not available or was not in the English language. Reviews, protocols, conference papers, and case reports were also excluded. Therefore, only original researches with original data on animal models or human patients exploring any kind of fasting regimes on gut microbiota were included in the present study.	Design of the study, loss of the study

*^1^PICOS, participants, intervention, comparator, outcome, study type.*

## Results

Twenty-eight articles were included in the qualitative synthesis. The characteristics of the evaluated studies and their results, including the results of animal studies and human studies, are listed in Tables 2, 3. The following are the results of animal studies and then the results of human studies.

**TABLE 2:** Summary of the animal studies investigating the effects of fasting on gut microbiota.

Study	Dietary restriction regimen	Study model	Gut microbiota variations induced by dietary restrictions	Potential health benefits	Microbiota analyzing methods
Shi et al. (21)	IF for 4 days in two cycles	Hypertensive rat	*Lactobacillus* and *Bifidobacterium* abundance increased in the IF group than control.	Rats in the IF group had significantly lower blood pressure than control group.	Shotgun sequence analysis of the microbiota and untargeted metabolomics
Zhang et al. (42)	(1) Fed *ad libitum*, (2) 30% CR, (3) 5:2 IF regimen	7-week-old C57BL/6 male mice	30% CR led to a significant increase in the *Lactobacillus*, and significant reduction in the Bacteroidetes. 5:2 IF regimen led to increase in the *Bacteroides*, *Alloprevotella* and significant reduction in the *Lactobacillus*.	IF group consume more energy than *ad libitum* and CR groups in the first 4 days after refeeding. Both of the CR and IF group had lower body weights, white adipose tissue and serum cholesterol than *ad libitum* group.	16S rRNA gene sequencing
Liu et al. (14)	Four groups: control (C), intermittent fasting (F), melatonin (M), and intermittent fasting plus melatonin (MF)	Male C57BL/6J mice	The F and M groups had significantly lower alpha diversity than the MF group. Increase in the abundance of *Lactobacillus*, *Ruminococcus*, and *Akkermansia* in the F group than control group. Reduction in the abundance of *Helicobacter*, *Prevotella*, and *Parasutterella* in the F group than C group.	There was no difference between the groups in the cumulative food intake. IF group had lower body weight, serum glucose and TG than control or melatonin groups.	16S rRNA gene v3–v4 amplicon
Deng et al. (23)	*Ad libitum* (AL) group or an IF group for 30 days	Male C57BL/6J mice	IF did not change the bacterial community richness Reduction in the Firmicutes to Bacteroidetes (F/B ratio) and relative increase in the *Allobaculum* abundance.	Weight was significantly reduced in the fasting group, but the cumulative energy intake was not different. IF reduced liver steatosis and lipid metabolisme.	16S rDNA gene amplicon sequencing
Li et al. (28)	*Ad libitum* control group or intermittent fasting groups.	C57BL/6JLvri mice	There were not significant differences between two groups in alpha diversity Mice in the 16 h fasting had increased level of *Akkermansia* and decreased level of *Alistipes*.	Cumulative food intake was not changed in the 12 h fasting but changed in the 16 and 20 h fasting.	16S rRNA gene amplicon sequencing
Park et al. (43)	IF vs. ketogenic diet	Male Sprague Dawley rats: Alzheimer’s disease (AD) model	In the IF group than keto group: Clostridiales abundance decrease and Lactobacillales increase.	IF than keto improved memory function.	16S rRNA amplicon sequencing
Kim et al., (44)	Fasting: the ruminal fluids feeding and 24 h after fasting	Three ruminally cannulated Holstein steers	Reduced abundance of *Anaerovibrio lipolytica*, *Eubacterium ruminantium*, *Prevotella albensis*, *Prevotella ruminicola*, and *Ruminobacter amylophilus*.	Increase in the gas, ammonia, and microbial protein production.	Denaturing gradient gel electrophoresis and quantitative polymerase chain react
Cignarella et al. (22)	In the IF mice, food pellets were provided or removed at 9 a.m. each day. Control group had unrestricted access to food	Mice	Lactobacillaceae, Bacteroidaceae, and Prevotellaceae families increased in the IF group. Fecal transplantation from mice in IF group to control, reduced the severity of EAE in this group.	IF reduced the differentiation of native T cells into T17 cells.	16S rRNA gene sequencing
Catterson et al. (45)	A 40-day course includes 2-day fed and 5 fasting days	Fruit flies (*Drosophila melanogaster*)	Reduced bacterial abundance in IF group than control Reduction in age-related pathologies and improved gut barrier function in the IF group.	Increases Stress Resistance, not changed cumulative food intake.	qPCR quantification of bacterial load
Beli et al. (12)	*Ad libitum* diet vs. intermittent fasting *ad libitum* diet as 24 h feeding-24 h fasting	db/db mice	Increased levels of Firmicutes and decreased Bacteroidetes and Verrucomicrobia in intermittent fasting group.	Glycated hemoglobin levels were not affected by the IF regimen, survival rate was significantly improved in the IF group.	16S rRNA sequencing with the MiSeq platform
Wei et al., (46)	Fasting diet with 30% restriction of calorie for 1 week	6-week-old male C57BL/ksJ-db	Increase in the Lactobacillaceae, Bacterioidaceae, and Prevotellaceae abundance.	Increase in the ketone production Decrease in the proinflammatory cytokines.	16 s rRNA sequencing
Bahl et al. (15)	3 days of food deprivation (fasting)	Farmed mink (Neovision vision)	The bacterial load and community structure within the mucus was not severely impacted by 3 days of fasting.	–	16S rRNA gene sequencing
McCue et al. (47)	21 days of fasting	Mice, quail, tilapia, toad, geckos	Alteration in Bacteriodetes, Firmicutes, Proteobacteria, Fusobacteria, and Verrucomicrobia.	Changes in distal intestine morphology.	16S rRNA sequencing
Sonoyama et al. (48)	96 h fasting compared to the control group	Male Syrian hamsters	Increase in the proportions of injured bacterial Cells Increase *Akkermansia muciniphila*, a mucin degrader, in fasting group Clostridia increased in the fed group	Reduction of total SCFA concentration in the fasted group than fed group.	16S rRNA clone library and species specific real-time quantitative PCR

*AD, Alzheimer’s disease; CR, calorie restriction; IF, Intermediate fasting; SCFA: short chain fatty acid.*

**TABLE 3:** Summary of the human studies investigating the effects of fasting on gut microbiota alterations.

References	Fasting model	Study type/duration	Study population	Results
Su et al. (49)	1 month of intermittent fasting	Longitudinal physiologic data in 2 cohorts, sampled in 2 different years	Healthy non-obese young and middle-aged men	Ramadan-associated intermittent fasting increased microbiome diversity and was specifically associated with upregulation of the Clostridiales order–derived Lachnospiraceae
Mohammadzadeh et al. (39)	Hour time restricted feeding intervention (8-h feeding window/16-h fasting window)	Before/after the cross-sectional study	Healthy adult volunteers (*n* = 30)	Butyrate significantly increases, the gut Bacteroides and Firmicutes increased by 21 and 13% after Ramadan.
Gabel et al. (40)	A daily 8-h time restricted feeding (8-h feeding window/16-h fasting window) for 12 weeks	Pilot study/12 weeks	Adults with obesity (*n* = 14)	Gut microbiota phylogenetic diversity remained unchanged.
Maifeld et al. (50)	Ramadan fasting	Clinical trial	Healthy subjects (*n* = 30)	Fasting alters the gut microbiome, impacting bacterial taxa and gene modules associated with short-chain fatty acid production.
Maifeld et al. (50)	5-days with a daily nutritional energy intake of 300–350 kcal/day, derived from vegetable juices and vegetable broth, followed by a modified Dietary Approach to Stop Hypertension diet	Randomized-controlled bi-centric/12 weeks	Patients with Metabolic Syndrome (*n* = 32–31)	Fasting alters the gut microbiome, impacting bacterial taxa and gene modules associated with short-chain fatty acid production.
Lilja et al. (52)	Buchinger fasting: 250 kcal/day for 5 days	RCT	154 healthy adults	↑ Distribution of Proteobacteria, ↓ Firmicutes/Bacteroidetes ratio fasting mimetic
Guo et al. (41)	“Two-day” modified IF	Clinical trial, 8 weeks	Adults with Metabolic Syndrome (*n* = 39)	Changes in gut microbiota communities, increase the production of short-chain fatty acids, and decrease the circulating levels of lipopolysaccharides.
He et al. (53)	Water-only fast or juice fast for 7 days	Intervention pre-post design	16 healthy individuals, age: 18–40 years	Water-only fasting changed the bacterial community, ↑ more homogenous gut microbiomes, ↓ *Fusobacterium*. ↓ Colorectal cancer
Ali et al. (38)	Ramadan fasting	Cohort	Healthy adult participants (*n* = 34)	↑ *Klebsiella*, *Faecalibacterium*, *Sutterella*, *Parabacteroides*, and *Alistipes* ↓ *Coprococcus*, *Clostridium_XlV*, and *Lachnospiracea*
Balogh et al. (55)	Buchinger fasting protocol followed by DASH diet	RCT/5 days	Control (*n* = 36), fasting (*n* = 35)	*↑ Clostridial Firmicutes* ↓ *Butyrate producers*
Ozkul et al. (37)	Ramadan fasting	Pilot study/29 days	Healthy adult participants (*n* = 9)	*Butyricicoccus, Bacteroides, Faecalibacterium, Roseburia, Allobaculum, Eubacterium, Dialister*, and *Erysipelotrichi* were significantly enriched genera after the end of Ramadan fasting.
Mesnage et al. (56)	Buchinger fasting (daily energy intake of about 250 kcal and an enema every 2 days	Clinical study/10-day	Healthy men (*n* = 15)	Decrease in the abundance of Lachnospiraceae and Ruminococcaceae increase in Bacteroidetes and Proteobacteria (*Escherichia coli* and *Bilophila wadsworthia*).
Remely et al. (57)	A fasting program with laxative treatment for 1 week followed by a 6-week intervention with a probiotic formula	One week	Overweight people (*n* = 13)	Fasting group had higher abundance of *Faecalibacterium prausnitzii*, *Akkermansia*, and *Bifidobacteri*

## Experimental Studies

Various animal studies have evaluated changes in the gut microbiome following a variety of fasting diets. Most animal studies on this interaction have been conducted in the past 5 years. Liu et al. in an experimental study compared the effect of intermediate fasting (IF) with melatonin administration on clinical variables and changes in the intestinal microbiome. They found that IF compared to the control group led to a significant increase in the abundance of *Lactobacillus*, *Ruminococcus*, and *Akkermansia* strains. Also, they found a significant reduction in the abundance of *Helicobacter*, *Prevotella*, and *Parasutterella* in the IF group (14). In another study on farmed mink (Neovision vision), the gut microbiota load and diversity showed no change after 3 days of fasting. Firmicutes were as the major phylum in the gut of these animals, however the Proteobacteria and Fusobacteria also were seen in another study (15). The rapid movement of food through the gastrointestinal tract may not allow enough time for bacterial metabolism to provide an environment that is suitable for growth of anaerobes (16).

Beli et al., evaluated the effects of long-term IF on gut microbiome, retinopathy and prolongs survival in db/db mice. The animals were fed *ad libitum* (AL) before the IF was initiated at 4 months of age. The db/db mice in the intervention group were then exposed to IF daily for up to 7 months. Microbiome analysis revealed increased levels of Firmicutes and decreased levels of Bacteroidetes and Verrucomicrobia in the IF group than control. Compared to the db/db mice on AL feeding, microbiome changes in the fasting group were associated with an increase in the gut mucin, goblet cell number, villi length, and reductions in plasma peptidoglycan (12). It has been reported in the previous studies that higher Firmicutes to Bacteroidetes ratio is associated with obesity (16, 17), as well as improve energy harvesting capacity (18). In this study, researchers used measurement of plasma peptidoglycan levels as an indicator of damage to the blood-brain barrier, and the results showed that IF regimen reduced plasma peptidoglycan levels and improved blood-brain barrier integrity. It has also been shown that a decrease in peptidoglycan concentration following IF is consistent with a reduction in endotoxemia (12). Therefore, fasting diets effect on weight loss through changes in the gut microbiota diversity and number, as well as peptidoglycan production. Gut microbiota involves major energy metabolic processes (19). Some studies have found a significant association between intestinal dysbiosis and energy dysmetabolism-induced chronic diseases such as diabetes, metabolic syndrome, and obesity (20). The positive results of the IF regimen on animal models with hypertension have also been shown in some studies (21).

Another part of animal studies has evaluated the effect of fasting on intestinal microbiome in animal models of neurodegenerative diseases. Cignarella et al. evaluated the effects of IF on gut microbiome and clinical symptoms of animal models of multiple sclerosis (MS), which named experimental autoimmune encephalomyelitis (EAE). They found that IF led to a significant improvement in the gut bacteria richness, enrichment of the Lactobacillaceae, Bacteroidaceae, and Prevotellaceae families and enhanced antioxidative microbial metabolic pathways. The results of this study also showed that the IF reduced the differentiation of native T cells into T17 cells, which secrete proinflammatory cytokines, and, conversely, increased the differentiation into regulatory T cells. Interestingly, the results of this study showed that fecal microbiome transplantation from mice under the fasting diet to mice with EAE ameliorated the symptoms, which could indicate the positive effect of the fasting diet (22).

On the other hand, some studies have shown that IF cause weight loss, reduce lipid peroxidation, and hepatic steatosis on obese mice through changes in microbial profile. Also, it has been reported in this study that IF led to a significant increase in the intestinal flora community diversity [Firmicutes to Bacteroidetes (F/B ratio) and relative increase in the *Allobaculum* abundance] (23). Increasing the abundance of Firmicutes following fasting diets can increase the production of short-chain fatty acids (SCFAs). SCFAs have the ability to increase the integrity of gut barrier, strengthen the immune system, reduce weight and insulin resistance (24). Moreover, fasting diets effect on the α-diversity (richness) and β-diversity (variety) of gut microbiota (12). Some pre-clinical studies have shown that IF could increase β-diversity, but the results on the effect of fasting diets on α-diversity are contradictory (12, 22, 25). Seven months IF on mice gut microbiota increased β-diversity compared in animals (12). Furthermore, weight loss introduced as the important and effective factor on α-diversity of gut microbiota (22), however it varies greatly during the day and dependents to dietary content (26, 27).

On the other hand, some studies have evaluated the effect of fasting diets on gut microbiota changes. Li et al. evaluated the effect of 12, 16, or 20-h fasting diets on the gut microbiome for 1 month. The results of this study showed that the composition of the gut microbiome changed in all types of fasting diets. At genus level, 16 h fasting led to increased level of *Akkermansia* and decreased level of *Alistipes*, but these effects disappeared after the cessation of fasting. No taxonomic differences were identified in the other two groups (28). In some previous findings, an increase in *Akkermansia* strains has been associated with metabolic benefits such as a reduction in the severity of fatty liver and intestinal inflammation (29). Increased levels of *Alistipes* can also exacerbate gut inflammation (30, 31).

Given that different metabolites are produced by the gastrointestinal microbiome, some other studies have evaluated these metabolites produced by microbiota following fasting diets. It has been reported an increased plasma levels of some metabolites such as tryptophan, serotonin, tryptophan, various bile acids, propionate, and acetate following the administration of fasting diets in animal samples (25, 32, 33). These results have also been confirmed in some human studies (34). Changes in the production of some metabolites can affect processes such as inflammation in the body. For example, some preclinical studies have shown that fasting diets exert inhibitory effects on the biosynthesis pathways of lipopolysaccharides by altering the intestinal microbiome. Lipopolysaccharides are among the major constituents of the outer membrane of Gram-negative bacteria, and studies have shown that increased production of lipopolysaccharides can induce toll like receptor-4 (TLR-4). TLR4 represents a key receptor on which both infectious and non-infectious stimuli converge to induce a proinflammatory response (35).

## Human Studies

According to the positive results of pre-clinical studies, in recent years, various human studies have evaluated the association between intestinal microbiome and fasting. In some human studies, fasting diet of Ramadan type on intestinal microbiome has been evaluated (13, 36–39). The duration of fasting time was 12–18 h per day in these studies. The results of these studies mainly showed changes in the intestinal microbiome following adherence to Ramadan fasting, some of which are mentioned below. In a clinical study in 2021, Mohammadzadeh et al. evaluated the effect of Ramadan fasting on serum levels of butyrate, intestinal microbiome and lipid profile. The results of this study showed that the serum level of butyrate in the fasting group increased significantly after 1 month. There was also a significant increase in the bacteroides and filminus strains in the intervention group (39). In another study, which conducted on Pakistani and Chinese participants, researchers evaluated the effect of a 29-day Ramadan fasting on alpha and beta diversity. The results of this study showed that the population of some bacterial strains such as Bacteroidetes and Firmicutes increased in the Pakistani population following fasting, however no noticeable changes were observed in the Chinese population. In addition, it has been reported that fasting in both populations affects beta diversity. Moreover, lower levels of genus *Coprococcus* observed after Ramadan fasting suggesting that fasting could have implications on health. On the other hand, fasting could also have harmful effects on health (38). A study of two cohort data showed that following a Ramadan-associated IF increased microbiome diversity and was specifically associated with upregulation of the Clostridiales order–derived Lachnospiraceae. In fact, the fasting diet in this study increased the expression of butyric acid-producing Lachnospiraceae. These alternations were independent of living area, body weight and diet composition and disappeared again when fasting was stopped (13). Various studies have shown that changes in the intestinal microbiome cause changes in physiological functions and reduce energy intake. Thus, human microbiome can be an effector for physiologic effects of IF (13). In another preliminary study, it was found that following the Islamic fasting diet caused significant changes in the intestinal microbiome, so that the number of *A. muciniphila* and *B*acteroides *fragilis* group members increased, however, *Lactobacillus* spp. and *Bifidobacterium* spp. remained relatively unchanged perhaps due to low fiber intake (36).

In addition to Ramadan fasting, some studies have examined the effect of restricted feeding in a form of IF on the intestinal microbiome. One of the major problems seen in these studies is the low sample size. Therefore, it is difficult to generalize the results of these studies to large populations. Gabei et al. in a pilot study evaluated the effect of fasting in a form of IF on the intestinal microbiome in adults with obesity. They found that IF led to a significant weight loss. Baseline evaluation of fecal microbiome by 16 S rRNA (ribosomal ribonucleic acid) gene sequencing showed that the predominant strains included Firmicutes and Bacteroidetes. However, at the end of 12 weeks of fasting diet, no significant change was observed in the abundance and distribution of dominant bacterial strains (40). However, the results of some other studies were inconsistent with this study. Guo et al. in a RCT study were evaluated the effects of 8 weeks of “2-day” modified IF in patients with metabolic syndrome. The results of this study revealed that 8 weeks of “2-day” modified IF led to a significant reduction in fat mass, oxidative stress, inflammatory cytokines, and improved vasodilatory parameters. On the other hand, the results of this study showed that following the 8 weeks of “2-day” modified IF caused a significant change in the composition of the intestinal microbiome, increased the production of SCFA and decreased lipopolysaccharide levels (41).

## Conclusion

In this review study, we evaluated the effects of Ramadan and non-Ramadan IF on gut microbiome. The results of most animal and human studies indicate the positive effects of fasting on the composition and structure of the gut microbiome. In addition to the positive role of fasting on the composition and abundance of intestinal microbiome, in some studies, other positive results have been observed following the observance of fasting regimes. Positive alterations in gut microbiota, such as overexpression of *A. muciniphila*, *B. fragilis*, *Bacteroides*, and butyric acid-producing Lachnospiraceae, were found to be associated with improved health indicators and decreasing disease development during Ramadan fasting. However, factors such as the duration of fasting diets, the presence of chronic diseases and obesity can affect the results. Considering the role of intestinal microbiome changes in the management of various diseases, future studies, especially clinical studies, should evaluate the impact of fasting regimes, especially Ramadan, on the management of various diseases through changes in the intestinal microbiome.

## Author Contributions

MR and ER: conception and design, and systematic search. SM and JH: screening and data extraction. MR and RT: manuscript writing. All authors contributed to the article and approved the submitted version.

## Conflict of Interest

The authors declare that the research was conducted in the absence of any commercial or financial relationships that could be construed as a potential conflict of interest.

## Publisher’s Note

All claims expressed in this article are solely those of the authors and do not necessarily represent those of their affiliated organizations, or those of the publisher, the editors and the reviewers. Any product that may be evaluated in this article, or claim that may be made by its manufacturer, is not guaranteed or endorsed by the publisher.
